# 15-Year-old with neglected recto-vestibular fistula in western Uganda: a case report

**DOI:** 10.1186/s13256-021-02717-5

**Published:** 2021-02-25

**Authors:** Felix Oyania, Meera Kotagal, Martin Situma

**Affiliations:** 1 Department of Surgery, Pediatric Surgical Unit, Mbarara University of Science and Technology, Mbarara, Uganda; 2 Department of Surgery, Kabale University School of Medicine, Kabale, Uganda; 3 Cincinnati Children’s Medical Centre, Cincinnati, USA

**Keywords:** Anorectal malformation, Krickenbeck, Bowel function, Teenage presentation

## Abstract

**Background:**

Teenage and late presentation of anorectal malformations are not uncommon in developing world. Some of the reasons for late presentation include but not limited to illiteracy, poverty, lack of awareness, and limited trained pediatric surgeons. In rural areas, neonates with ARMs are considered cursed and are marginalized.

**Case:**

15-Year-old African girl (a munyankole by tribe in Uganda) from western Uganda presented at 15 years of life with colostomy and uncorrected anorectal malformation. Never went to school due to social stigma.

**Conclusion:**

Due to limited number of trained pediatric surgeons in most of African Countries, many children in addition to living with a colostomy or untreated malformation, may also be undiagnosed with chronic constipation. Improved awareness and advocacy would promote early presentation and treatment.

## Introduction

Anorectal malformations (ARMs) are common congenital anomalies with a reported incidence of approximately 1 in 5000 live births [1]. They have been classified according to Krickenbeck classification [2, 3]. The most common type in females is vestibular fistula [4–8]. Teenage and late presentation of anorectal malformations are not uncommon in the developing world. Some of the reasons for late presentation include lack of awareness, limited trained pediatric surgeons, illiteracy, poverty and child negligence. In some rural areas in Uganda, neonates with ARMs are considered cursed and are marginalized. These patients usually present because of marital reasons as the girl approaches puberty [8–12].

## Case presentation

**Fig. 1:**
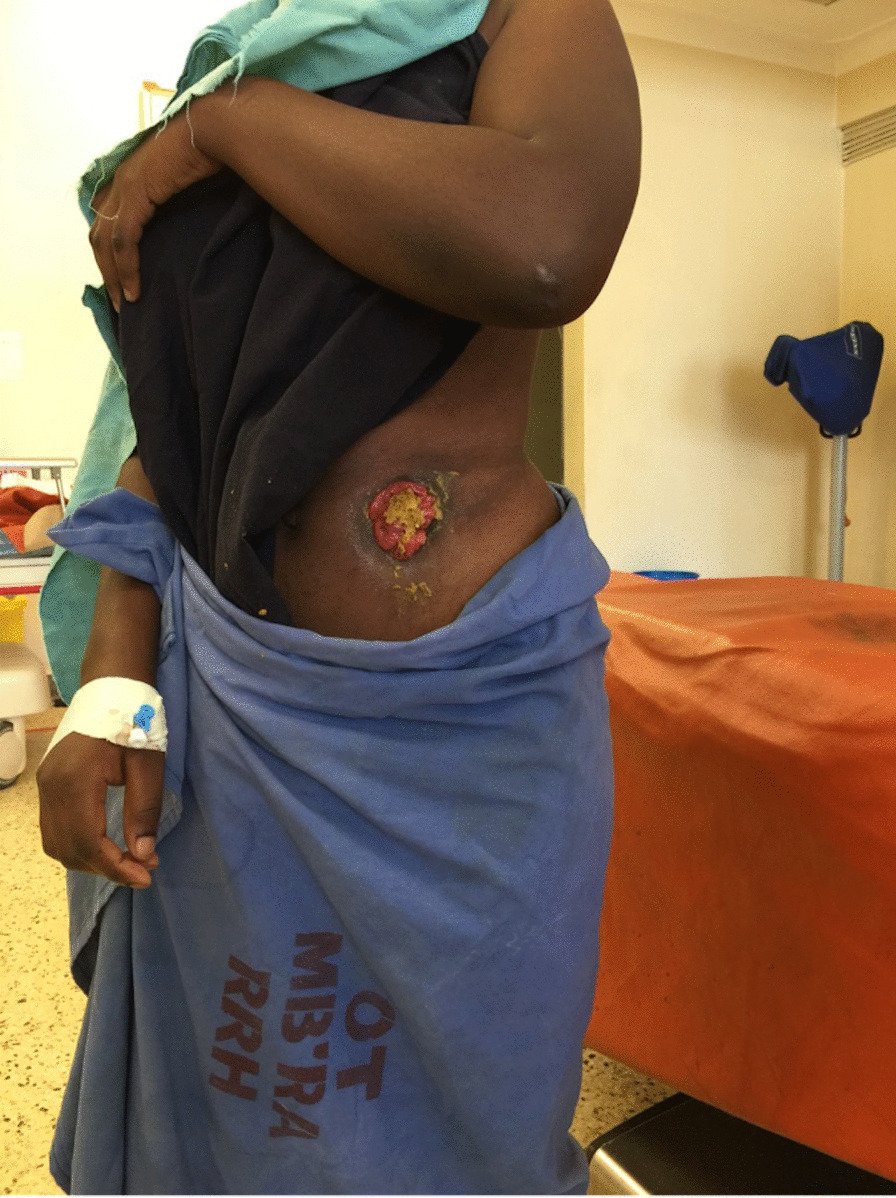
Before posterior sagittal anorectoplasty

A 15-year-old African female (a munyankole by tribe in Uganda) presented with a sigmoid colostomy and an uncorrected ARM. Colostomy was placed when she was 2 months old. The operation was performed at what was then the only pediatric surgical unit in the country in Kampala, the capital city of Uganda. She shortly lost her parents to unknown illnesses, was moved to the village and has been raised by her maternal grandmother. Her grandmother thought the condition was unrepairable. She never went to school due to social stigma and has been marginalized in her village and considered cursed. A well-wisher who feared that she would not be considered suitable for marriage because of her colostomy brought her to our facility (Fig. 1).

At the time of presentation, she was a well-nourished teenage girl with evident secondary female characteristics, weighing 53 kg, Abdominal examination was normal with a well-functioning double barrel sigmoid colostomy. Her blood work was normal. She was noted on perineal exam to have a vestibular fistula.

**Fig. 2:**
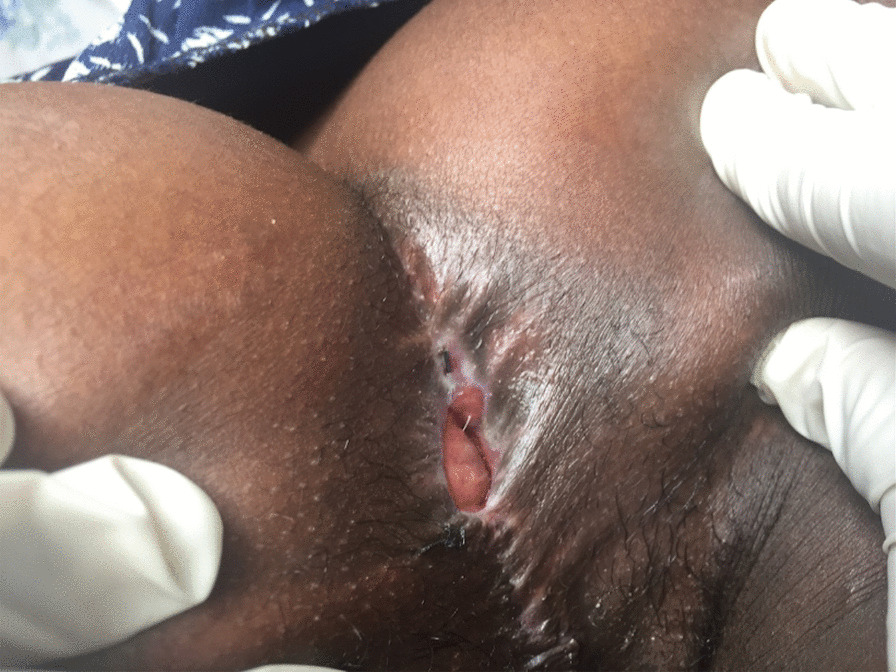
The Neoanus before colostomy closure

Posterior sagittal anorectoplasty (PSARP) was performed and the neoanus was calibrated to 17 mm at the end of repair. She underwent serial anal dilatation according our ward protocol, achieved anal size for age calibrated at 18 mm Hegar (Fig. 2). After 6 weeks colostomy was taken down. She is being followed in the clinic. Her short term follow-ups of three months so far revealed normal functional outcome.

Ethical approval was obtained from Mbarara University research ethics committee #30/05-20. Written informed consent form was obtained from the legal guardian and assent from the child.

## Discussion

Teenage and late presentation of anorectal malformations is not rare in developing world; however, they are correctable congenital malformations. Some of the reasons for late presentation include but not limited to illiteracy, poverty, lack of awareness, child negligence, lack of social support, and limited trained pediatric surgeons. In rural areas, neonates with ARMs are considered cursed and are marginalized [8–14. Our patient lived with colostomy because of lack of awareness of her grandmother when she lost her parents. Some patients usually present because of marital reasons as the girl approaches puberty their parents or guardians bring them to the hospital [8,15].

### Impact of late presentation

Delayed presentation and diagnosis of ARMs may lead to complications such as constipation, delayed and altered surgical management, recurrent genital and urinary tract infections, infertility, inadequate weight gain, difficult toilet training increased parental anxiety, and functional and psychological problems for adult patients [16–18]. Other studies have reported complications of constipation, anal excoriation, occasional soiling, mucosal prolapse and sexual abnormalities [19, 20]. Often times there is significant dilation of rectum/colon due to distal obstruction from a small fistula opening, making repair more difficult and decreasing functionality of bowel.

Despite late presentation, patients with vestibular fistula after corrective surgery are able to conceive and carry the pregnancy to term, and deliver children through normal vaginal route [20, 21]. Therefore, community awareness through support groups and stoma care groups might help improve presentation, and as a result, the outcome.

Believing that community awareness could be increased in this fashion, we created community led support group for anorectal malformation in the Mbarara region of western Ugandan which was officially launched on June 12, 2019. This has substantially improved awareness in western Uganda with more patients presenting with colostomies for definitive repair.

## Conclusion

Due to limited number of trained pediatric surgeons in most African countries, as well as low community awareness about the possibility of definitive repair, many children live with a colostomy or untreated malformation. As a result, they often have undiagnosed chronic constipation and fecal incontinence. These result in significant social ostracization and limited ability to go to school. Improved awareness and advocacy would promote early presentation and treatment.

## Data Availability

Supporting data is available upon request.

## Abbreviations

ARMs: Anorectal malformations
PSARP: Posterior sagittal anorectoplasty
